# Preparation, Characterization and Release of Urea from Wheat Gluten Electrospun Membranes

**DOI:** 10.3390/ma5122903

**Published:** 2012-12-18

**Authors:** Daniela Denisse Castro-Enríquez, Francisco Rodríguez-Félix, Benjamín Ramírez-Wong, Patricia Isabel Torres-Chávez, María Mónica Castillo-Ortega, Dora Evelia Rodríguez-Félix, Lorena Armenta-Villegas, Ana Irene Ledesma-Osuna

**Affiliations:** 1 Department of Chemical-Biological Sciences (DCQB), University of Sonora, Hermosillo, Sonora C.P. 83000, Mexico; E-Mail: ladany_305@hotmail.com; 2 Department of Food Research & Graduate Program (DIPA), University of Sonora, Hermosillo, Sonora C.P. 83000, Mexico; E-Mails: bramirez@guaymas.uson.mx (B.R.-W.); pitorres@guayacan.uson.mx (P.I.T.-C.); iledezma@guayacan.uson.mx (A.I.L.-O.); 3 Research Department in Polymers & Materials (DIPM), University of Sonora, Hermosillo, Sonora C.P. 83000, Mexico; E-Mails: monicac@guaymas.uson.mx (M.M.C.-O.); dora@polimeros.uson.mx (D.E.R.-F.); powerl14@hotmail.com (L.A.-V.)

**Keywords:** wheat gluten, electrospinning, prolonged-release system, urea

## Abstract

Homogeneous and thin porous membranes composed of oriented fibers were obtained from wheat gluten (WG) using the electrospinning technique. SEM micrographs showed an asymmetric structure and some porosity, which, in addition to a small thickness of 40 μm, are desirable characteristics for the membranes’ potential application in release systems. The membranes were loaded with urea to obtain pastilles. FT-IR and DSC studies confirmed the existence of interactions via hydrogen bonding between urea and WG proteins. The pastilles were studied as prolonged-released systems of urea in water. The release of urea during the first 10 min was very fast; then, the rate of release decreased as it reached equilibrium at 300 min, with a total of ≈98% urea released. TGA analysis showed that the release system obtained is thermally stable up to a temperature of 117 °C. It was concluded that a prolonged-release system of urea could be satisfactorily produced using WG fibers obtained by electrospinning for potential application in agricultural crops.

## 1. Introduction

Urea is an inexpensive solid nitrogen fertilizer used for agricultural production [1]. However, its use involves the loss of the fertilizer by leaching and volatilization, which may be exacerbated by high temperature and humidity under environmental conditions [2]. Peña *et al.* [3] studied the nitrogen cycle using isotopic techniques (^15^N) and reported nitrogen losses of up to 90%, emphasizing that the greatest losses occur by leaching, which is closely linked to water management. These losses of fertilizer lead to poor harvests and great economic loss for producers, as well as potential environmental contamination. An alternative solution for the efficient use of nitrogen fertilizer is the use of devices that allow for the slow or controlled release of this nutrient [4]. 

Recently, investigations have been carried out to obtain prolonged-release systems of fertilizers, and different materials that can be used in these systems have been reported [5,6,7,8,9]. However, the proposed application requires a material that is 100% natural, biodegradable, inexpensive and highly available. These characteristics are necessary to prevent soil contamination and obtain a release system at low cost. Wheat gluten (WG) is a raw material that meets these characteristics: It is renewable, biodegradable and available in large quantities at competitive prices [10,11]. The composition of WG is complex and includes glutenin and gliadin proteins [12]. Glutenin is composed of a group of polymeric gluten proteins. The molecules consist of glutenin subunits linked together through disulfide bonds; these subunits are classified into two groups: high-molecular-weight and low-molecular-weight glutenin subunits (HMW-GS and LMW-GS) [13]. Gliadins are monomeric proteins that are classified, in order of decreasing mobility in acidic polyacrylamide gel electrophoresis, as α-, β-, γ- or ω-gliadins [14]. Disulfide bonds play an important role in the solubility of these proteins; gliadins are soluble in alcoholic solutions, while glutenins are soluble in acidic solutions [15,16]. Gluten proteins are insoluble in water; thus, they can be used as a matrix for the prolonged-release systems of urea.

A desirable characteristic of the prolonged-release systems of substances is a high surface area [17]. Using the electrospinning technique, materials with this property can be obtained. The electrospinning technique is a practical method used to produce new materials on the micro- to nano-meter scale and can be applied to all soluble or meltable polymers [18]. Using this technique, interesting results have been reported regarding the production of fibers from WG using 1,1,1,3,3,3-hexafluoro-2-propanol (HFIP) as the solvent and various additives [19,20,21]. However, HFIP is volatile and corrosive. HFIP vapors can cause severe respiratory problems and immediate, irreversible eye damage. Thus, alternative solvent systems must be investigated. Moreover, the application of materials obtained from WG using the electrospinning technique has not been reported. The morphological characteristics of the materials obtained by electrospinning allows for several different applications, including the use of the products as release materials [22]. Using the electrospinning technique, WG-based materials for use as prolonged-release systems of urea can be obtained, providing a potential solution to the problem of loss by leaching of this fertilizer in agricultural crops. Considering all of the above information, the main objective of this work was to prepare and characterize a prolonged-release system of urea from WG by the electrospinning technique.

In the present article, we report the preparation and characterization of membranes created from WG using common solvents via electrospinning and the results of a preliminary evaluation of the membranes as prolonged-release systems of urea.

## 2. Results and Discussion

### 2.1. Obtaining Membranes by Electrospinning

Table 1 shows the different conditions studied to obtain membranes from WG by electrospinning. The optimal conditions for obtaining membranes by electrospinning were an 8% w/v concentration of WG, an ethanol/2-mercaptoethanol (solution 8) solvent system, a distance of 10 cm between the needle and the collector plate, a solution flow rate of 0.01 mL h^−1^ and an applied voltage of 15 kV. These conditions were established according to the macroscopic characteristics of the material obtained. The homogeneity of the material formed was mainly considered. A homogeneous and thin membrane with a thickness of 40 μm was obtained. This small thickness is desirable for the membrane’s potential application in a prolonged-release system. 

**Table 1:** Electrospinning conditions for the preparation of membranes from wheat gluten.

Solution	Distance collector (cm)	Flow rate (mL h^−1^)	Voltage (kV)	Characteristic
1	15	0.1	15	Only drops
1	15	0.1	20	Only drops
2	15	0.03	15	Only drops
2	15	0.03	20	Only drops
3	15	0.01	15	Thin layer
3	15	0.01	18	Thin layer
3	15	0.03	15	Thin layer
3	15	0.03	18	Thin layer
4	15	0.01	15	Thin layer
4	15	0.03	15	Thin layer
4	15	0.05	15	Only drops
5	15	0.01	15	Thin layer
6	15	0.01	15	Thin layer with drops
6	10	0.01	10	Thin layer with drops
7	10	0.05	20	Thin layer with drops dispersed
8	10	0.01	15	Thin layer and homogeneous

Various studies conducted to obtain WG films using mainly the casting technique have recently been reported [23,24,25,26,27,28]; the use of extrusion [29] and thermo-compression [30,31] has also been considered. However, there are no reports indicating the production of WG films with a thickness as small as those observed in this study. Iglesias *et al.* [30] used the thermo-compression technique to obtain WG films with a thickness of 230 µm. The thickness of these films is nearly six times higher than that obtained in this study using the electrospinning technique. On the other hand, Chiou *et al.* [31] studied the effect of temperature and the addition of cold sulfuric acid on the thickness of films obtained from WG using the method of thermo-compression and reported film thicknesses in the range of 150 to 600 µm, corresponding to four to 15 times the thickness found in our study. These results suggest that the electrospinning technique is the best option for obtaining a thin WG membrane. 

### 2.2. Microscopic Characteristics

**Figure 1:**
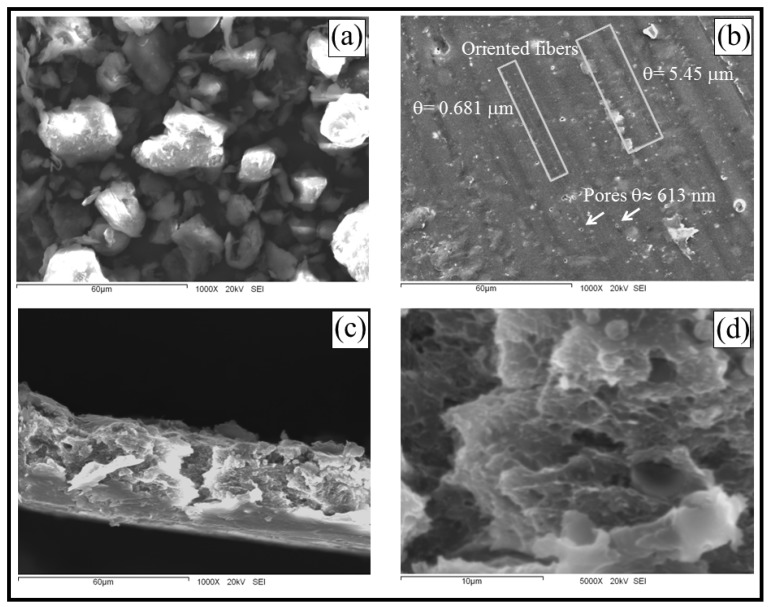
Scanning electron microscopy (SEM) micrographs of (**a**) WG powder; (**b**) WG membrane surface; and (**c**,**d**) cross section of WG membranes at different magnifications.

The microscopic characteristics of the materials obtained were evaluated using scanning electron microscopy (SEM). Figure 1a,b show SEM micrographs of a WG powder and the surface of a WG membrane, respectively, both at a magnification of 1000×. Amorphous granules measuring 5 to 30 µm for the WG powder were observed. 

In this study, WG membranes composed of oriented cylindrical fibers and featuring some porosity were observed; the fiber diameter varied from 0.683 to 5.45 µm, and the pore diameter was ≈613 nm (Figure 1b). An estimation of the membrane porosity was obtained from digitized SEM micrographs of the membrane surface (Figure 1b), the average porosity was estimated to be 6.05%. The porosity observed in the fibrous membranes favors their potential application in prolonged-release systems. The size of the granules of WG powder, fiber diameter, pore diameter and estimation of the membrane porosity were determined by image analysis using the software Image Tool 3.0 Final. Figure 1c,d show cross-sectional SEM micrographs of WG membranes at magnifications of 1000× and 5000×, respectively. An asymmetrical structure was observed. This characteristic is desirable for increasing the encapsulation efficiency of the released material, which in this study is urea.

### 2.3. Fourier Transform-Infrared Spectroscopy, FT-IR

**Figure 2:**
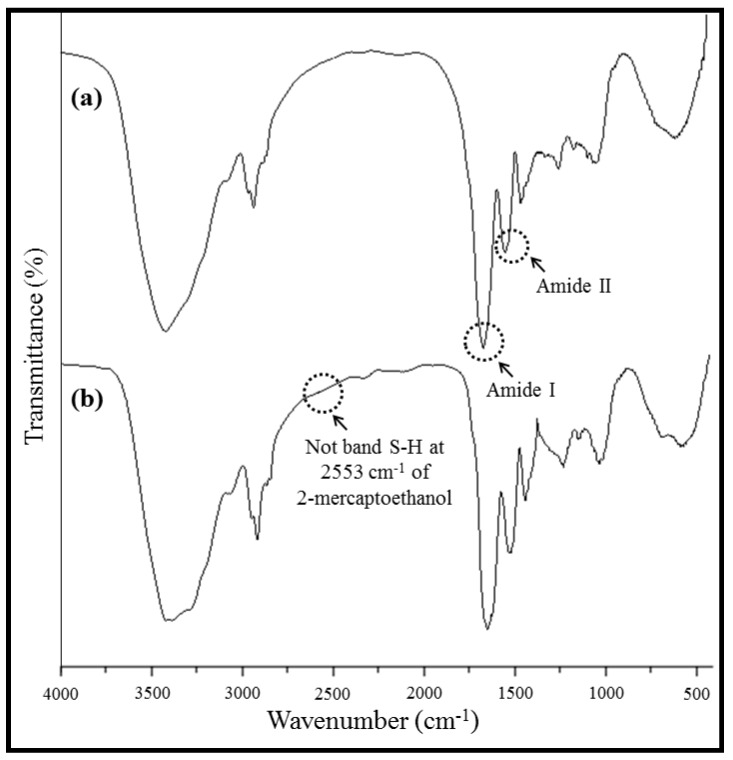
Fourier transform-infrared spectroscopy (FT-IR) of (**a**) WG powder and (**b**) WG membranes obtained by electrospinning.

Figure 2a,b shows the FT-IR spectra of a WG powder and WG membrane, respectively. Both FT-IR spectra showed the following bands: A band of strong intensity at 3413 cm^−1^ attributed to the stretching of the O–H bonds of amino acids present in the proteins of wheat gluten; a band of medium intensity at ≈2928 cm^−1^, which corresponds to the stretching of the –CH_2_– group, and two bands of different intensities, a band of strong intensity at ≈1655 cm^−1^ associated with the band vibration frequency of amide I. This band is related to the vibrational stretching of the C=O group. The second band of medium intensity at ≈1540 cm^−1^ is related to the band vibrational frequency of amide II. This band corresponds to the deformation of the N–H group [32,33]. In the FT-IR spectra of the WG membranes, no band was observed at 2553 cm^−1^, which would correspond to the deformation of the –SH group and be representative of 2-mercaptoethanol [34]; thus, these spectra indicate the absence of any remnant of this reagent in the material obtained by electrospinning. The FT-IR spectrum of the WG membranes is similar to the spectrum of the WG powder, which indicates that the electrospinning technique does not affect the chemical composition of WG.

**Figure 3:**
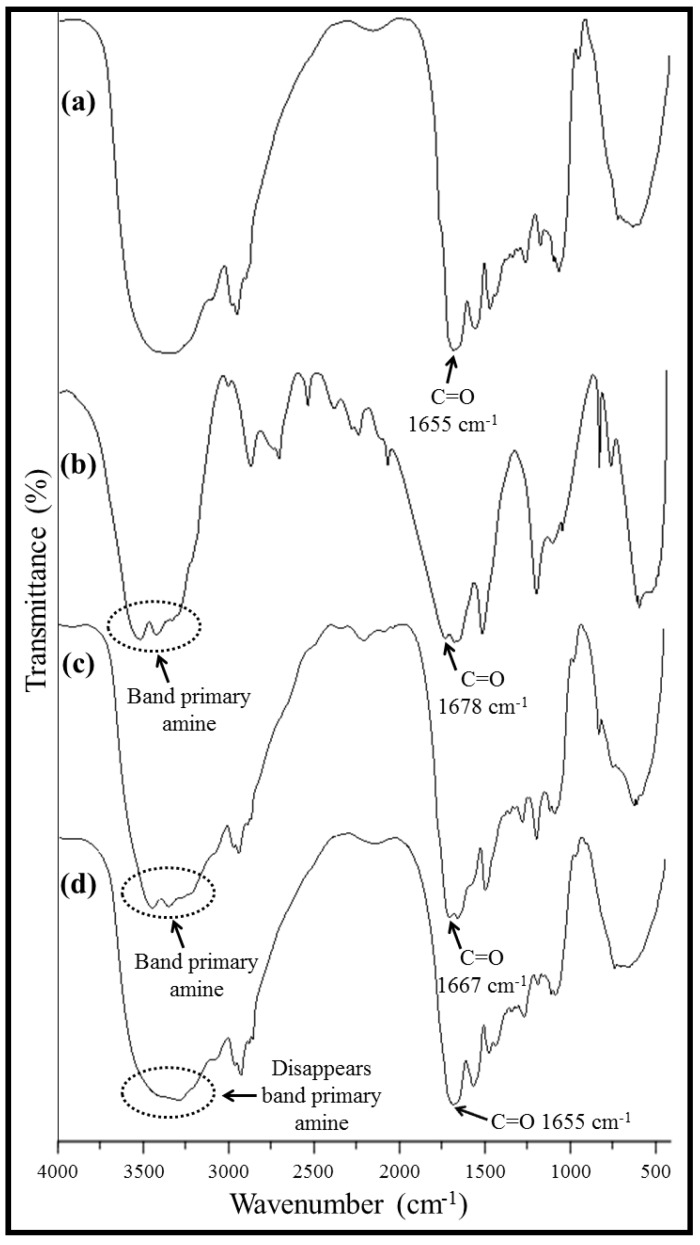
FT-IR spectra of (**a**) pastille composed of WG membranes loaded with water; (**b**) urea powder; (**c**) pastille composed of WG membranes loaded with urea; and (**d**) pastille composed of WG membranes loaded with urea after the release test. All pastilles were freeze-dried before analysis.

Figure 3a shows the FT-IR spectrum of a pastille obtained from WG membranes loaded with water and freeze-dried; the same bands that were discussed for the WG powder and WG membranes were observed, including a band of strong intensity at 1655 cm^−1^ associated with the vibrational stretching of the C=O group (amide I) and a band at ≈1540 cm^−1^ related to the vibrational frequency of the N–H group (amide II). This result indicates that the chemical composition of WG is not affected by the processing conditions used. 

Figure 3b shows the FT-IR spectrum of urea powder. The band characteristic of the stretching of the N–H bond corresponding to primary amine was observed. This band is composed of two peaks corresponding to each vibration of the N–H bond of primary amine: one at 3442 cm^−1^ and the other at 3348 cm^−1^. Another two bands of strong intensity appeared at 1678 cm^−1^ for the C=O group and 1624 cm^−1^ for the vibrational stretching of the N–H group. A band of medium intensity observed at 1465 cm^−1^ was attributed to the vibrational stretching of the C–N bond of urea. Figure 3c,d shows the FT-IR spectra of pastilles obtained from WG membranes loaded with urea before and after conducting a release test, respectively. Both pastilles were freeze-dried before being analyzed by infrared spectroscopy. Before the release test, the bands characteristic of a primary amine were observed in the same regions of the urea powder spectrum, indicating that urea was present in the pastille. Moreover, shifts in the position of the carbonyl band, a 12 cm^−1^ shift for the WG pastille (Figure 3a) and an 11 cm^−1^ shift for urea (Figure 3b), were observed. This shift is attributable to a possible interaction via hydrogen bonding between the amino group of the proteins and the carbonyl group of the urea and *vice versa*. This interaction suggests the effective adsorption of urea onto the WG membranes. Finally, after the release test (Figure 3d), the band corresponding to the vibration of the N–H bond of primary amine, which is characteristic of urea, disappeared. Moreover, the carbonyl band returned to the initial position of 1655 cm^−1^, corresponding to the pastille without urea (Figure 3a). These results suggest the complete release of urea.

### 2.4. Thermogravimetric Analysis, TGA

Table 2 shows the loss in weight of the WG powder, WG membranes and pastille composed of WG membranes loaded with urea, respectively, as a function of temperature. The three materials show similar behavior. The first weight loss occurred approximately at 100 °C, which was attributed to the loss of moisture. The WG powder presented the highest weight loss, 7%, at this temperature, while the WG membranes loaded with urea presented the lowest weight loss, 2%; this result is attributed to the freeze-drying of these samples. For the pastille composed of WG membranes loaded with urea, an additional weight loss of 19% in the range of 117 to 207 °C was observed; this loss is attributed to the degradation of urea, indicating that the release system obtained can be used up to a temperature of 117 °C. Finally, all samples were degraded by approximately 100% at 600 °C.

### 2.5. Differential Scanning Calorimetry, DSC

Table 2 shows the *T*g values obtained by DSC. The 9 °C decrease in the value of *T*g for WG membranes obtained by electrospinning, compared with WG powder, is attributed to the reduction of the disulfide bonds between the protein chains of wheat gluten. This reduction is due to the action of 2-mercaptoethanol, which was used to reduce the molecular weight of the protein chains and, thus, to dissolve the WG powder in ethanol. However, the *T*g value of the pastille composed of WG membranes loaded with urea increased by 29 °C relative to the *T*g value of the membrane without urea. This increase is attributed to hydrogen bond interactions between the carbonyl group of the urea and amino groups of WG and *vice versa*. These interactions generate a more rigid material, similar to a cross-linked material, therefore increasing the *T*g value [33]. These results corroborate the possible hydrogen bonding interaction proposed by FT-IR studies (Figure 3). 

**Table 2:** Thermogravimetric analysis (TGA) and Glass transition temperature (*T*g) obtained by differential scanning calorimetry (DSC).

Sample	TGA		DSC
	Temperature (°C)	Loss in weight (%)	*T*g (°C)
WG powder	100	7	149
	160–350	49	
	350–600	37	
WG membranes	100	4	140
	100–357	50	
	390–568	40	
Pastille from WG membranes loaded with urea	100	2	169
	117–207	19	
	207–350	38	
	350–600	42	

WG = Wheat gluten

### 2.6. Urea Release Kinetic

The electrospun-membrane mass required to trap 1 mL of a 1 M urea solution was 0.15 g, and membranes loaded with urea were placed in molds to obtain pastilles. Under these conditions, only a suspension was formed with the unprocessed WG, which cannot be used in the release process. The entrapment efficiency of urea in electrospun membranes was calculated by gravimetry and was found to have an 86% of efficiency corresponding to 51.63 mg of urea. This value is higher than reported by Chen *et al.* [35]. They prepared a new type of slow-release membrane-encapsulated urea fertilizer with starch-g-PLLA as biodegradable carrier materials, using the technique of casting. In their study, they found an encapsulation efficiency of 53% for unmodified starch and 81% for starch-g-PLLA. These results suggest that the electrospinning technique is more suitable than the casting technique to obtain membranes for encapsulate substances; this can be attributed to the low thickness of the membrane (40 µm) obtained by electrospinning.

**Figure 4:**
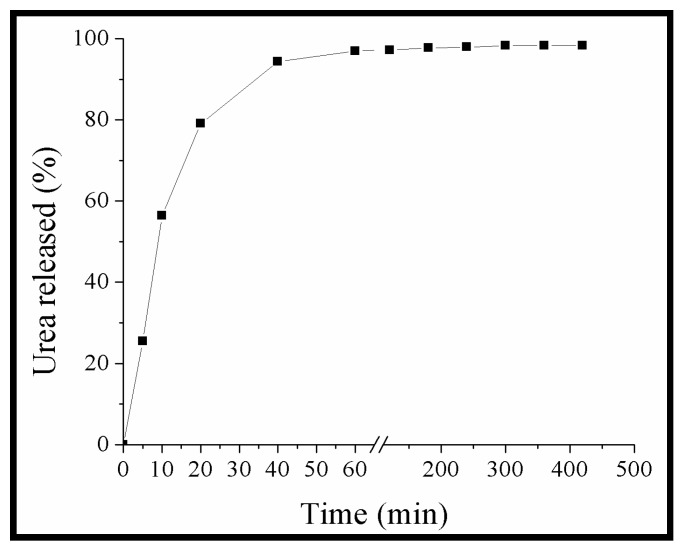
Release kinetics of urea in water at 25 °C from pastille composed of WG membranes loaded with urea.

Figure 4 shows the release kinetic of urea in water for pastilles obtained from WG membranes loaded with urea. The release of urea during the first 10 min was very fast (56% of the original urea concentration was released), *i.e.*, a burst effect was observed [36]. Similar results were reported by Mulder *et al.* [37]. They developed an economically feasible and biodegradable slow-release coating for urea. For this purpose, commercially available lignin (soda flax lignin, Bioplast) and different hydrophobizing agents or cross-linkers on urea granules were used. They reported ≈80% of urea release at 10 min for the coating with Bioplast, and ≈65%, 53% and 44% for Bioplast coating containing 2-2-phosphinicobis-butanedioic acid, formaldehyde and alkenyl succinic anhydride, respectively, and reached a release equilibrium ≈25 min. In our study, after 10 min, the release rate decreased until equilibrium was reached in approximately 300 min (5 h), with a total of ≈98% urea released. On the other hand, the results obtained in this experiment are important, because if we place the same amount of urea without any pretreatment (as is done with agricultural crops) under the same conditions of the release test, the urea is immediately dissolved. Using urea in this way has been reported to lead to losses in agricultural crops of up to 90% [3] Therefore, the use of the prolonged-release system of urea produced can be a potential solution to the problem of urea loss by lixiviation in agricultural crops. 

### 2.7. Empirical Model for the Urea Release Kinetic

In this study, an overall evaluation of the kinetics and mechanism of urea release from electrospun membranes was done according to the exponential model developed by Ritger and Peppas [38]:

$$\frac{M_{t}}{M_{\infty }}=kt^{n}$$

were *M*_t_ and *M*_∞_ are the mass of urea released at time *t* and ∞, respectively, *k* is a kinetic constant, and *n* is the diffusion exponent that can be related to the drug transport mechanism. For a thin hydrogel, when *n* = 0.5, the drug release mechanism is Fickian diffusion. When *n* = 1, Case II transport occurs, leading to zero-order release. When the value of *n* is between 0.5 and 1, anomalous transport is observed [39,40]. Then, according to the value obtained of *n* = 0.81, a mechanism of anomalous transport (Non-Fickian) is proposed for the release of urea in water, this mechanism occurs when the diffusion and relaxation rates are comparable, *i.e.*, the urea release depends on two simultaneous processes: water migration into the device and urea diffusion through electrospun membranes. The value of *k* was 0.07; this value is similar to that reported in our previously published paper for release of amoxicillin from hydrogels composed of poly(acrylamide) and poly(γ-glutamic acid) [36].

## 3. Experimental Section

### 3.1. Materials

The materials used were commercial wheat gluten, Roquette; acetic acid, glacial, Sigma; acetone, Faga Lab; ethanol, J.T. Baker; 1-propanol, Sigma Aldrich; 2-propanol, J.T. Baker; 2-mercaptoethanol, Sigma Aldrich; urea, Fermont; and a urease Berthelot kit, Randox. All reagents were used as received.

### 3.2. Methods

#### 3.2.1. Solutions Preparation

Table 3 presents the conditions for the preparation of the main solutions that were used for electrospinning. The concentration (%, w/v) of wheat gluten was varied, and different common solvent systems were used. 2-mercaptoethanol was used to reduce the disulfide bonds between the proteins of wheat gluten. The solutions were obtained using constant magnetic stirring for one hour.

**Table 3:** Conditions for the preparation of solutions from wheat gluten.

Solution	Solvent system	Concentration (w/v)
1	Acetic acid 70% ^a^/Ethanol 70% ^a^ 1:1	8%
2	Acetic acid 70% ^a^/Ethanol 70% ^a^ 1:1	12%
3	Acetic acid 70% ^a^/2-propanol 70% ^a^ 1:3	12%
4	Ethanol 80% ^a^/Acetone 80% ^a^ 1:1	12%
5	1-propanol 70% ^a^/2-mercaptoethanol 19:1	12%
6	Acetic acid 70% ^b^/Ethanol 70% ^b^/2-mercaptoethanol 95:95:10	12%
7	Acetic acid 80% ^a^/Ethanol 50% ^a^/2-mercaptoethanol 95:95:10	12%
8	Ethanol 50% ^a^/2-mercaptoethanol 19:1	8%

^a^ in water; ^b^ in acetone at 70% ^a^.

#### 3.2.2. Fibrous Membrane Preparation

Five variables in the technique of electrospinning were controlled: the concentration of the solution, solvent system, applied voltage and flow rate solution and distance between the needle and the collector plate. The WG solution was transferred to a plastic syringe with a 5 mL capacity and a 0.8 mm diameter needle. Using a KD Scientific syringe pump, the flow rate solution was varied from 0.01 to 0.1 mL h^−1^. A high voltage in the range of 10–20 kV was applied to the wheat gluten solution using a high-voltage power supply (Spellman, model CZE 1000R). Finally, the distance between the needle and the collector plate was varied, using distances of 10 or 15 cm. A square plate of aluminum measuring 10 cm × 10 cm was used as the collector.

#### 3.2.3. Characterization

The morphology of the membranes was evaluated using a JEOL 5410LV scanning electron microscope operated at 20 kV. The samples were gold-sputtered prior to the SEM examination. FT-IR spectroscopy was used to study the interactions between the urea and the material obtained. Dried samples were mixed with KBr powder, and pastilles were made. FT-IR characterization was performed using a Perkin-Elmer Spectrum GX FTIR spectrometer. Thermal analyses (TGA and DSC) were carried out on an SDT 2960 simultaneous DSC-TGA instrument from TA Instruments. Samples of 5 mg were warmed to 600 °C at a heating and cooling rate of 10 °C min^−1^ under a flow of 23 mL min^−1^ of synthetic air as purge gas.

#### 3.2.4. Loading of Urea in the Wheat Gluten Films

WG membranes obtained via electrospinning were loaded with urea, and pastilles were made. The loading of urea into the membranes was carried out by the swelling equilibrium method. The membranes were immersed in 1 mL of a 1 M urea solution containing 60.06 mg of urea, the amount required to give a concentration of 0.0025 M in irrigation, which is the maximum concentration that can be reached during the first irrigation of a wheat crop. The membrane mass required to trap the total solution was 0.15 g. The membranes loaded with urea were placed in molds with a 12 mm diameter and 5 mm thickness; these were freeze-dried for 48 h in a Labconco Freezone 4.5 unit under a vacuum pressure of 0.15 mBar and a temperature of −46 °C in the collector. Pastilles were thus obtained.

#### 3.2.5. Urea Release Kinetic Experiments

For experiments concerning the prolonged release of urea, the dried pastilles were placed in a glass with 400 mL of distilled water. The glass was covered to prevent the evaporation of water and constantly agitated at 25 °C in a shaker-type incubator (model Max Q 4000, Barnstead). Aliquots of 10 µL were taken in vials at different times. The amount of urea released was determined using the Berthelot method (Randox kit) by ultraviolet visible spectroscopy using a Perkin-Elmer Lambda 20 UV-Vis spectrophotometer. All tests were performed in triplicate.

## 4. Conclusions

The optimal conditions for the preparation of membranes from WG using common solvents via the electrospinning technique were determined. Membranes with very small thicknesses not previously reported using other techniques were obtained. The morphological characteristics of the membranes obtained indicate that they can be used as release systems, and the urea release test demonstrated the membranes’ potential application as prolonged-release systems of urea, due to their porosity and the hydrogen bonding interactions formed between urea and WG proteins. These interactions were confirmed by FT-IR and DSC studies, and a TGA analysis revealed that the release system obtained is thermally stable up to a temperature of 117 °C. It is concluded that a prolonged-release system of urea composed of WG membranes obtained by electrospinning can be satisfactorily produced for potential application in agricultural crops.
